# Analysis of Financial Crowding Out in the Iraqi Economy for the Period 2004 – 2022

**DOI:** 10.12688/f1000research.173509.1

**Published:** 2026-02-10

**Authors:** Ahmed Jabbar, Basim Dikheel

**Affiliations:** 1 Financial and banking sciences, Al Iraqia University, Baghdad, Baghdad Governorate, Iraq; 2 Finance and banking, University of Baghdad Al-Waziriya Campus College of Administration and Economy, Baghdad, Baghdad Governorate, Iraq

**Keywords:** : Financial Crowding out, Government Credit, Private Credit, Parkinson's Law, Financial Expulsion, Financial Attraction.

## Abstract

The Iraqi economy’s performance is impacted by the rivalry between public and private investment, which raises the country’s debt and reduces private investment, which in turn slows economic growth. As a result, the subject of crowding out is closely tied to both the public and private sectors and how they affect the country’s economy. To determine whether financial crowding out has occurred and whether there is a reciprocal relationship between government credit and private credit, this research focused on confirming the existence of financial crowding out through the levels of government credit and private credit in Iraq. Imbalances in the dynamic term of the agreement result in the long-term achievement of the difference, and the existence of integration implies the existence of a different economic connection. In both the short and long terms, there was a considerable inverse relationship between government credit and private credit, according to trends and conventional tests like the direction of the error limit mitigation (VECM) and (Wald). Due to the rise in government credit at the expense of private credit in the Iraqi economy, it produces outcomes that are comparable to those of a crowding-out model for financial credit.

## 1. Introduction

The Iraqi economy is considered a rentier economy, characterized by financial mismanagement, chronic budget deficits, and the heavy influence of government spending, coupled with weak public revenues due to the rentier nature of these revenues. Since 2004, the Iraqi economy has experienced successive increases in public spending at a rate exceeding the growth in public revenues. This is due to the transformation of the state apparatus into a large employment agency for the workforce, according to Parkinson’s Law, which stipulates that employment increases beyond the actual need, resulting in a decline in the role of the private sector in employment and investment. This has led to financial crowding out of the private sector. ^1^ The increase in public spending has resulted in a financial deficit in the general budget and an increase in government borrowing to cover obligatory expenses, or what are called governing or sovereign expenses, which are expenses that cannot be deferred, with a lack of direction or allocation of this borrowing towards investment areas, which may be a factor of attraction, not a factor of repulsion, for private investment, according to Keynesian theory. ^2^ This study concluded that there is a two-way cointegration relationship between government credit and private credit. The existence of cointegration implies the presence of an economic relationship between them, and that short-term imbalances in the relationship indicate the potential for achieving a long-term balance. Standard models and tests, such as the vector error correction model (VECM) and the Wald test, revealed a significant causal relationship in the opposite direction between government credit and private credit in both the short and long terms. ^3^ These results suggest the existence of financial crowding out of private credit due to increases in government credit in the Iraqi economy, at the expense of private credit. ^4^

The study aims to shed light on the relationship between the government and the private sector in the Iraqi economy and to demonstrate the extent of government intervention and uncontrolled expansionary financial policies.

### 1.1 Theoretical background

Financial crowding out of the private sector by the government occurs when competition for financial resources within the banking and financial system intensifies, resulting in reduced opportunities for private investment. The government enters the competition for financial resources when its funding channels shift from these resources to meet the government’s need for public spending. ^2^ Financial crowding out is defined as the government’s exclusion of the private sector from financial resources and the negative impact of a state’s general budget deficit on private investment. ^5^

Primary economic schools differed on the subject of financial crowding out. The Keynesian school explained that government investment attracts private investment and does not displace it (crowding in private investment), provided that government investment is directed towards establishing the infrastructure of the national economy, reducing production costs for the private sector, and increasing profit levels through the gateway of aggregate demand. Meanwhile, the monetary theory confirmed that government investment crowds out private investment (financial crowding out), as the balance between savings and investment is achieved through the equilibrium interest rate. Since government investment maximizes the general budget deficit, and financing this deficit through the issuance of low-value nominal bonds means a rise in the interest rate and an increase in investment costs for the private sector, the contraction or displacement of this sector has been achieved due to the budget deficit resulting from the rise in the rate of government investment. Here, government investment replaces private investment, thus achieving financial crowding out. ^2, 3^ The Ricardian equilibrium theory also explains the effect of changing interest rates on crowding out the private sector. This crowding out is linked to the method of financing public spending. If public spending is funded through public debt instead of taxes, this financing will have both direct and indirect effects on the economy. The direct effect is characterized by a direct, immediate rise in interest rates and an increased cost of investment for the private sector. ^6^ Here, financial crowding out of the private sector is achieved. The indirect effect is a future-oriented effect, characterized by a decline in consumption levels for the private sector and an increase in the volume of private savings, as individuals anticipate a future rise in taxes to offset the public debt incurred to finance past public spending. Here, crowding out also occurs due to the decline in private consumption and the rise in private savings. The effect of this on aggregate demand, which is considered the main source of achieving profits and raising production levels. ^4, 7^ While the monetary school asserts that the state’s role in crisis management is undesirable and that it lacks the capacity to overcome it, the state’s presence actually deepens the economic crisis, as it interferes with economic stability. The only way to resolve the crisis is to grant the private sector complete freedom. However, during economic cycles, the private sector may not be financially and economically displaced. Instead, what occurs is the private sector’s withdrawal from participating in the development of the national economy, as the government intervenes to address economic cycles and correct imbalances in the production structure. This is what happened during the Great Depression and other economic crises, when pessimistic expectations prevailed, leading to the private sector withdrawing and allowing the government sector to assume the primary decision-making role in managing economic and financial resources. ^5, 8^ The displacement of the private sector can be realized when the government addresses the negative real GDP gap, and the effect of increased government spending on GDP is observed. It is found that the increase in GDP is less than the expected increase in the dynamics of the government spending multiplier, which leads us to conclude that private spending was displaced when the government addressed the negative GDP gap. ^6– 8^

## 2. Materials and methods

**Figure 1.**
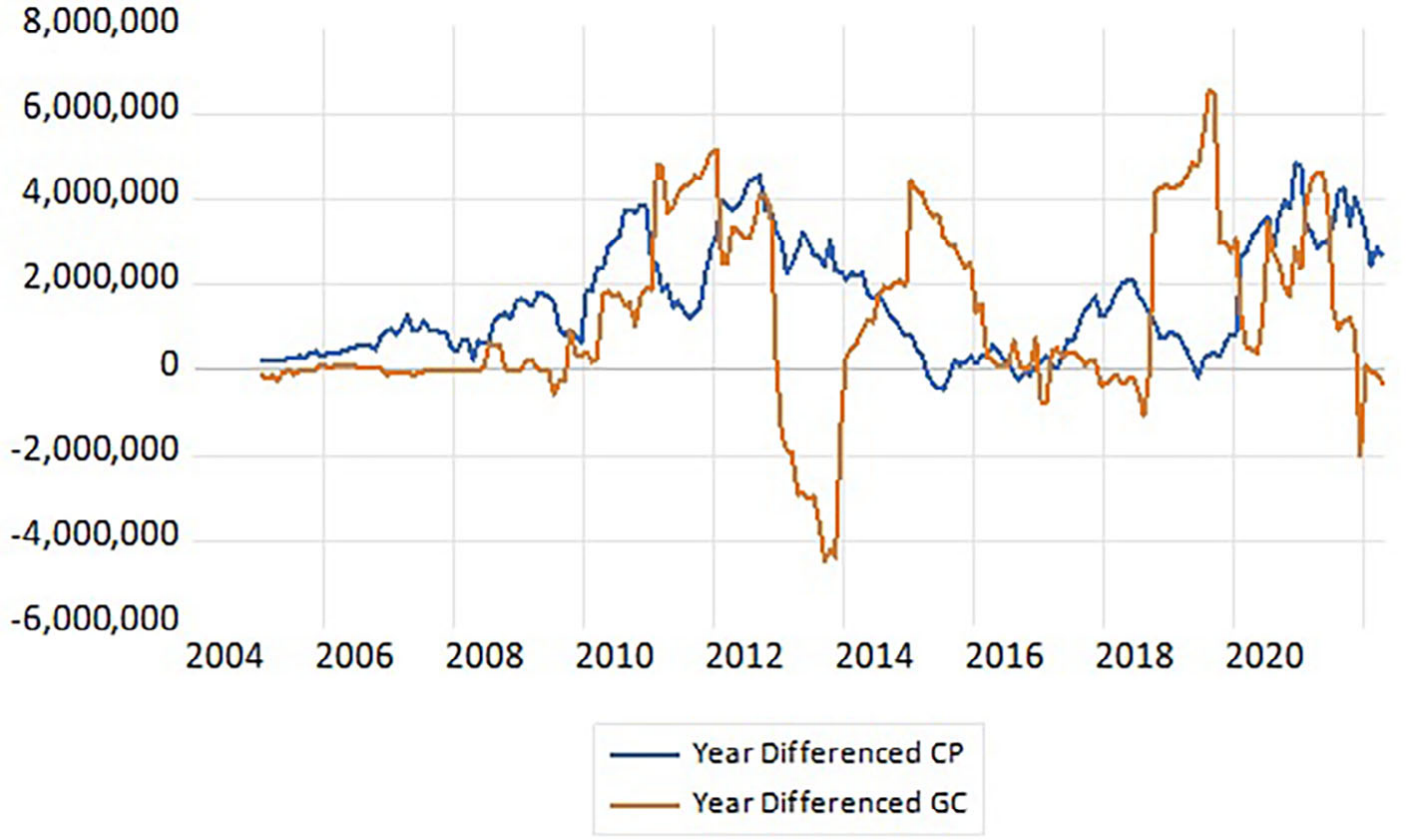
Positive and negative growth in government credit and private credit. **Source:** Prepared by the researcher based on the statistical program (E-views 12) and data from the monthly statistical supplement from the official website of the Central Bank of Iraq. ^9^

Descriptive analysis, graphic analysis, and statistical analysis using the statistical program (E-views 12) were used to analyze and measure financial crowding out in the Iraqi economy through government credit and private credit indicators taken from the Central Bank of Iraq for the period (2004-2022). ^9^ Many previous studies on the Iraqi economy have confirmed the existence of structural imbalances in the gross domestic product, structural imbalances in the general budget and mechanisms for managing public funds, in addition to the existence of unilateralism in the aspect of commodity exports abroad, which is summarized in the export of oil, which makes the Iraqi economy vulnerable to external economic fluctuations in the energy market. ^1, 2, 6, 10^ There is also a trend to increase public spending with every rise in oil prices and to finance this spending with public debt with every fall in oil prices, which results in a significant increase in government credit compared to private credit, which is basically weak due to the economic and political instability that Iraq is witnessing. ^11, 12^ Through the appendix of the monthly data for the series of government credit and private credit at the end of the research taken from the official website of the Central Bank of Iraq, ^9^ it is noted that the positive growth in government credit is met by negative growth in private credit on the one hand, and also noted the increasing growth in government credit is met by contradictory growth in private credit on the other hand, which gives the data a clear indication of the existence of a large area for government credit with a small area for private credit, meaning an economic and financial indication of the existence of financial crowding out in the Iraqi economy, ^6, 10– 12^ ( Figure 1). This positive growth in government credit, which is the opposite of the decreasing growth, is due to the state’s dominance over credits provided by commercial and government banks when there is a significant deficit in the general budget during economic and financial crises affecting foreign oil markets and political instability witnessed by the country, as many crises have affected the economic and financial performance of Iraq, specifically the performance of the state’s general budget, such as the financial crisis of 2008 and the global pandemic crisis of 2020-2021, and the repercussions of this crisis are still affecting the economies of many countries.

Through the relationship between government credit and private credit and the conclusion that can be drawn from the occurrence or non-occurrence of financial crowding out in the Iraqi economy, the standard model of the relationship between government credit and private credit is described. Based on economic theory, the inverse relationship between government credit and private credit has an economic significance for the occurrence of financial crowding out and its impact on the interest rate and the gross domestic product, as the increase in government credit at the expense of private credit due to the state’s general budget deficit leads to an increase in the interest rate and a decrease in private investment levels and its replacement by government investment. ^10– 12^ However, government investments are witnessing a significant decline, which leaves an impact on the level of economic growth and an increase in unemployment rates. Therefore, the nature and direction of the relationship between government credit and private credit will be verified within a series of standard tests. The researcher expects the existence of a strong inverse relationship between changes in government credit and private credit, and the linear equations ( eq. 1 and 2) are in the following form ^1^:

CP=f(GC) eq.1

CP=a−bGC+μ eq.2

**Where: CP** represents private credit as the dependent variable, **a** represents the constant term in the equation that represents the value of private credit in the absence of the effect of government credit, **b** represents the marginal slope that reflects the amount of marginal change in private credit as a result of changes in government credit, **GC** represents government credit, and **μ** represents the random error term that represents all other variables not included in the model.

## 3. Results and discussions

The vector error correction model (VECM) and the Wald test were applied, as indicated by the output of the standard program (EViews 12) for the time series of the variables included in the model. The stationarity of the time series was checked using the Augmented Dickey-Fuller (ADF) test and the Phillips-Perron (PP) test. The model was then presented, and the presence of cointegration was detected using the Wald Test and the Johansen-Juselius (1990) methods. ^1, 2, 6, 10^

### 3.1 Time series tests for private and government credit series

**3.1.1 Graphing method**

**Figure 2.**
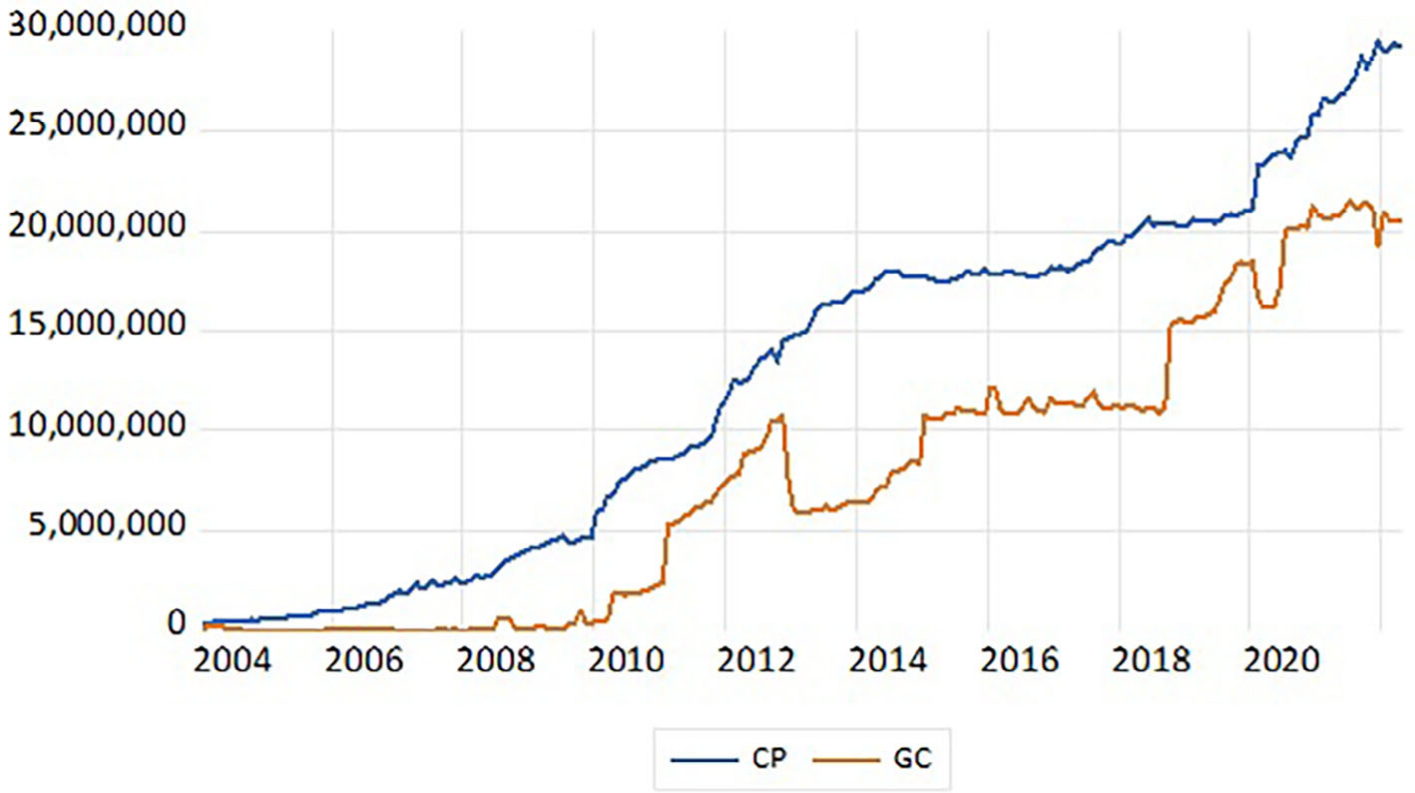
Static time series of government credit (GC) and private credit (CP) at level I(0). **Source:** Prepared by the researcher based on the statistical program (E-views 12).

The graph gives the time path of the economic variables under consideration the initial perception of the extent of the dormancy of the time series of any economic variable, as well as its ability to indicate a general upward or descending trend of the time series for this economic variable caused by the difference in the averages of the partial samples of the series as a whole and thus the lack of stillness of the time series, so one of the features of the dormancy of the time series at the graphic level is the stability of the arithmetic mean **E [y]** in each time period and Figure 2 of the government credit series GC and private credit CP at level I(0), it is noted from this graph the existence of an unstable upward trend in the government credit series and private credit in the sense of the instability of the arithmetic mean of the time series of the economic variables under research and this is an indication of the lack of stillness of the time series and the dormancy is verified through subsequent standard tests, the most famous of which is the test of Dickie Fuller and Phillips Perron. ^10, 11, 13^

**3.1.2 Dickey-Fuller Augmented test for government credit and private credit time series**

**Figure 3.**
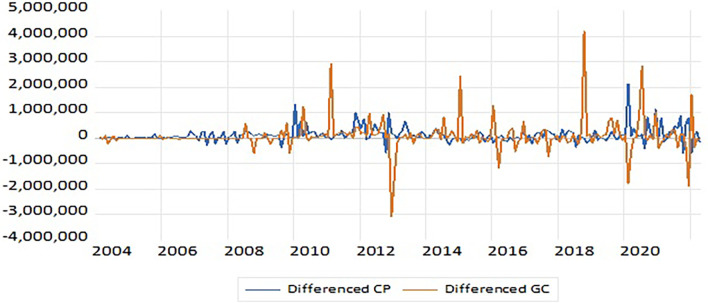
Dormancy of the time series of government credit GC and private credit CP in the first difference I(1). **Source:** Prepared by the researcher based on the statistical program (E-views 12).

To conduct the extended Dickey-Fuller test on the time series of economic variables, the time series of government credit GC and private credit CP for the first level and difference (with a fixed limit, a fixed limit and a general trend) was tested at the level of (5%) as shown in Table 1, so it became clear that the time series of government credit GC and private credit CP is not static at the general level, i.e. not integrated from the grade I(0) and after taking the first difference of the time series of government credit GC and private credit CP. The time series has become stable in the first difference, i.e. integrated of degree I(1). Figure 3 and does not suffer from the root of the unit, which means the possibility of rejecting the null hypothesis ( **H**_**o**_ **: B = 0**) that there is a problem of the root of the unit and accepting the alternative hypothesis ( **H**_**1**_ **:B ≠ 0**) that states the rest of the time series.

**Table 1.** D.F. test for the time series of government credit GC and private credit CP.

Time series	Constant	Constant and linear trend	Integration
Government credit GC	0.272413	-2.571431	I(0)
Test critical values	-3.460173	-4.000511	
	-2.874556	-3.430477	
	-2.573784	-3.138828	
Prob.*	0.9764	0.2939	
Government credit GC	-13.63328	-13.66821	I(1)
Test critical values	-3.460313	-4.000708	
	-2.874617	-3.430572	
	-2.573817	-3.138884	
Prob.*	0.0000	0.0000	
Private credit PC	1.423580	-1.568307	I(0)
Test critical values	-3.460173	-4.000511	
	-2.874556	-3.430477	
	-2.573784	-3.138828	
Prob.*	0.9991	0.8024	
Private credit PC	-15.43071	-15.61777	I(1)
Test critical values	-3.460313	-4.000708	
	-2.874617	-3.430572	
	-2.573817	-3.138884	
Prob.*	0.0000	0.0000	

**Source:** Prepared by the researcher based on the statistical program (E-views 12).

The economic significance of unit root tests for time series of economic model variables is to obtain reliable estimates of the relationship between the model variables, rather than spurious regression estimates, which are reflected in the absence of a significant or logical relationship between the model variables. At the same time, the coefficient of determination **R** ^**2**^ for the pseudo-relationship is very high, in addition to avoiding the occurrence of the problem of self-correlation between random variables in the model, as the unit root tests measure the stationarity of the time series of the model variables and show the significance of this stability between time series.

**3.1.3 Phillips-Perron Unit Root Test for GC and CP Time Series**

To conduct the Philips-Perron test on the time series of economic variables, the time series of government credit GC and private credit CP for the level and the first difference (with a fixed limit or a fixed limit and a general trend were tested at the level of 5%) according to the data of Table 2, so it became clear that the time series of government credit GC and private credit CP is not static at the general level, i.e. it is not integrated from the grade I(0) and that the time series of government credit GC and private credit CP became static after taking the difference The first has any integral of degree I(1) Figure 3 and does not suffer from the root of the unit, which means that the null hypothesis ( **H**_**o**_ **: B = 0**) that there is a unit root problem can be rejected and the alternative hypothesis **(H**_**1**_ **:B ≠ 0)** that states the time series is dormant.

**Table 2.** P.P. test for the time series of government credit GC and private credit CP.

Time series	Constant	Constant and linear trend	Integration
Government credit GC	0.234476	-2.669988	I(0)
Test critical values	-3.460173	-4.000511	
	-2.874556	-3.430477	
	-2.573784	-3.138828	
Prob.*	0.9742	0.2502	
Government credit GC	-13.59396	-13.62673	I(1)
Test critical values	-3.460313	-4.000708	
	-2.874617	-3.430572	
	-2.573817	-3.138884	
Prob.*	0.0000	0.0000	
Private credit PC	1.240933	-1.715641	I(0)
Test critical values	-3.460173	-4.000511	
	-2.874556	-3.430477	
	-2.573784	-3.138828	
Prob.*	0.9984	0.7413	
Private credit PC	-15.58616	-15.70909	I(1)
Test critical values	-3.460313	-4.000708	
	-2.874617	-3.430572	
	-2.573817	-3.138884	
Prob.*	0.0000	0.0000	

**Source:** Prepared by the researcher based on the statistical program (E-views 12).

### 3.2 Estimating the joint integration between government and private credit according to the joint integration methodology of Johanssen Joselius

The joint integration, or simultaneous association, means that there is an economic relationship between the variables, and these variables do not diverge significantly. There is a long-term balance relationship between them. ^10, 14^ After proving the Unit Root Test for the time series dormancy (Dicky-fuller Augmented test and PhiliPs – Perron) the time series dormancy in the first difference of government credit and private credit according to the extended Dicky-Fuller test and the Philips Perron test, now we will apply the co-integration methodology using Johansen-Juselius 1990 methodology, which is one of the best methods used to estimate the cointegration vector and confirm its unilateralism based on the trace test (trace λ) and the Maxium Eiganvalues test (max), which show the existence of a long-term equilibrium relationship between the economic variables of the research sampl. ^13, 14^ Table 3 shows the results of the trace test (trace λ) to analyze the long-term relationship between government credit and private credit, where the impact test shows that the calculated value (75.77) is greater than the critical value (15.49) at the level of 5%, and this means rejecting the null hypothesis ( **H**_**o**_ **: B = 0**) that there is no common integration vector between government credit and private credit and accepting the alternative hypothesis that there is one or more of the cointegration vectors, as well as the trace test (trace λ) reveals The existence of a second vector of joint integration, as the calculated value (23.89) is greater than the critical value (3.84) at the level of (5%). Thus, there is a relationship of two-way joint integration between government credit and private credit, and this is what makes us expect a causal relationship at least in one direction between the variables studied throughout the research period.

**Table 3.** Results of Johansen Joselius methodology for government and private credit.

	0.05	Trace		Hypothesized
Prob. **	Critical Value	Statistic	Eigenvalue	No. of CE(s)
0.0000	15.49471	75.77817	0.215293	None *
0.0000	3.841465	23.89507	0.105651	At most 1 *

	0.05	Max-Eigen		Hypothesized
Prob. **	Critical Value	Statistic	Eigenvalue	No. of CE(s)
0.0000	14.26460	51.88310	0.215293	None *
0.0000	3.841465	23.89507	0.105651	At most 1 *

Trace test indicates 2 cointegrating eqn(s) at the 0.05 level.

Unrestricted Cointegration Rank Test (Maximum Eigenvalue).

Max-eigenvalue test indicates 2 cointegrating eqn(s) at the 0.05 level.

**Source**: Prepared by the researcher based on the statistical program (E-views 12).

* denotes rejection of the hypothesis at the 0.05 level

** MacKinnon-Haug-Michelis (1999) p-values

* denotes rejection of the hypothesis at the 0.05 level

** MacKinnon-Haug-Michelis (1999) p-values.

Also Table 3 shows the results of the Maxium Eiganvalues test (λmax) to analyze the long-term relationship between government credit and private credit, where the effect test shows that the calculated value (51.88) is greater than the critical value (14.26) at the level of 5%, and this means rejecting the null hypothesis **(H**_**o**_ **: B = 0)** that there is no common integration vector between government credit and private credit and accepting the alternative hypothesis (r ≠ 0) or (r = 1) that there are one or more vectors of cointegration, as well as The Maxium Eiganvalues test (λmax) reveals the existence of a second vector for cointegration, as the calculated value (23.89) is greater than the critical value (3.84) at the level of (5%) Thus, there is a relationship for the joint integration between government credit and private credit, and this test represents a reinforcement of the results of the impact test in detecting the long-term relationship.

### 3.3 Causality testing according to a vector model to correct the error limit between government credit and private credit in the Iraqi economy for the period 2004-2022

The reason for choosing the Vector Error Correction Model test is to find out the existence of the long-term equilibrium relationship between the variables and the direction of this relationship. ^15– 17^ In the sense of what is the variable that causes the change of the other variable, more precisely, and from the standpoint of the research variables, does government credit affect private credit or vice versa, or is there a regressive relationship between the variables? The results of the error correction vector test show that there is a long-term equilibrium relationship in one direction from government credit to private credit during the study period, where the error limit parameter (T test) for private credit is negative and significant (-2.01198) and that the correction of error in the short term towards achieving balance in the long term is within two months (0.01 x 220 = 2.2), where the duration of the study is 220 months, as shown in Table 4.

**Table 4.** Vector error correction model for government and private credit.

	CointEq1	Cointegrating Eq:
	1.000000	DCP(-1)
	-10.50395	DGC(-1)
	(1.24978)	
	[-8.40466]	
	858132.4	C
D (DGC)	D (DCP)	Error Correction:
0.090439	-0.010989	CointEq1
(0.01070)	(0.00546)	
[8.45345]	[-2.01198]	
-0.183684	-0.851028	D (DCP(-1))
(0.12472)	(0.06368)	
[-1.47276]	[-13.3651]	
0.050404	-0.415298	D (DCP(-2))
(0.12526)	(0.06395)	
[0.40239]	[-6.49393]	
0.030288	-0.138294	D (DGC(-1))
(0.09387)	(0.04793)	
[0.32265]	[-2.88551]	
0.032249	-0.036691	D (DGC(-2))
(0.06964)	(0.03555)	
[0.46311]	[-1.03203]	
-470.9077	422.5426	C
(40935.0)	(20899.1)	
[-0.01150]	[0.02022]	

**Source**: Prepared by the researcher based on the statistical program (E-views 12).

Table 5 presents the conclusion derived from the error limit correction model, indicating an inverse relationship between government credit and credit for periods of reversion or time default. C(2) C(3) C(4) is negative and significant, and the value of C(1), which represents the value of correction of the error limit, is also a negative value (-0.010989) and significant (0.0455), and this indicates a substantial and equilibrium relationship from government credit to private credit and that the estimated model is substantial according to the value of F (38.95) and does not suffer from the problem of self-correlation between residuals or random variables, where the value of Darbin Watson (2.11) is greater than the upper limit of 1.60.

**Table 5.** Balance test schedule and correction of imbalance between government credit and private credit.

Prob.	t-statistic	Std. error	Coefficient	
0.0455	-2.011976	0.005462	-0.010989	C(1)
0.0000	-13.36508	0.063676	-0.851028	C(2)
0.0000	-6.493934	0.063952	-0.415298	C(3)
0.0043	-2.885513	0.047927	-0.138294	C(4)
0.3032	-1.032033	0.035552	-0.036691	C(5)
0.9839	0.020218	20899.07	422.5426	C(6)
-763.6296	Mean dependent var		0.481194	R-squared
421439.7	S.D. dependent var		0.468841	Adjusted R-squared
28.13543	Akaike info criterion		307147.9	S.E. of regression
28.22919	Schwarz criterion		1.98E+13	Sum squared resid
28.17331	Hannan-Quinn criterion		-3032.627	Log likelihood
2.115505	Durbin-Watson stat		38.95510	F-statistic
			0.000000	Prob(F-statistic)

**Source:** Prepared by the researcher based on the statistical program (E-views 12).

The existence of the short-term relationship between government credit and private credit can be verified within the series of standard tests, so to detect the existence of a short-term relationship between government credit and private credit, we resort to the Wald test to show the existence and absence of the short-term relationship, and the test results can be extracted in an integrated manner from the error limit correction model estimated in the table above. ^18– 20^ According to the Wald Test, as long as the Chi-square value, which amounted to (178.6253), is significant in terms of the probability value (0.0000), this indicates the existence of a short-term relationship between government credit and private credit, as shown in Table 6.

**Table 6.** Wald test for the short relationship between government and private credit.

Wald Test: Equation: Untitled			
Probability	df	Value	Test statistic
0.0000	210	-13.36508	t-statistic
0.0000	(1, 210)	178.6253	F-statistic
0.0000	1	178.6253	Chi-square
Null Hypothesis: C(2) = 0			
Null Hypothesis Summary:			
Std. Err.	Value	Normalized Restriction (= 0)	
0.063676	-0.851028	C(2)	
Restrictions are linear in coefficients			

**Source:** Prepared by the researcher based on the statistical program (E-views 12).

As shown in Table 7, VEC Granger Causality/Block Exogeneity Wald Tests, which is the Kanger test for causality to support the results of the VECM error limit correction vector model, and according to the results of this test, there is a one-way causal relationship from government credit to private credit, where the degree of significance of the relationship is less than 5%, i.e. reached (0.0067), and this is what reinforces the results reached by the researcher. ^19, 20^

**Table 7.** VEC Granger causality block exogeneity wald tests.

Dependent variable: D (DCP)			
Prob.	df	Chi-sq	Excluded
0.0067	2	10.02387	D (DGC)
0.0067	2	10.02387	All

Dependent variable: D (DGC)			
Prob.	df	Chi-sq	Excluded
0.0847	2	4.936145	D (DCP)
0.0847	2	4.936145	All

**Source:** Prepared by the researcher based on the statistical program (E-views 12).

## 4. Conclusions

1. The inverse relationship between government credit and private credit is a clear indication of the existence of financial crowding out.
2. Financial crowding out occurs in the Iraqi economy due to the nature of government credit, which is used to finance public consumer spending. This is contrary to the Keynesian school of thought.
3. The outdated approach to the state’s general budget and the dominance of political orientations over economic and financial decisions are the reasons for the creation of financial obligations that must be fulfilled and are considered an acquired right of the public, according to public choice theory. This means the occurrence of public borrowing and financial crowding out.
4. The independence of monetary policy from fiscal policy is behind the emergence of financial crowding out in the Iraqi economy.
5. The government’s continued borrowing makes the accumulated debt crisis more widespread through the fixed item in the general budget, debt service, due to the high interest rates on government credit. These high interest rates dissuade private investment.

In future work, efforts should be made to modify the state’s general budget methodology from an item budget to a program and performance budget, and to enhance the efficiency of fiscal policy to ensure that cases of sustainable general budget deficits do not occur. Increasing the private sector’s participation in economic development by creating infrastructure and reducing the costs associated with private investments. This is what the Keynesian school advocates, which ensures the occurrence of a state of financial attraction for the private sector instead of a state of financial expulsion—diversifying sources of income in the Iraqi economy by maximizing government and private investments, which in turn maximizes tax revenues, which ensures that a state of financial expulsion does not occur.

## Data Availability

The authors acknowledge that all data and supporting materials for the results or analyses in the research are available free of charge and can be accessed through the Central Bank of Iraq website at the following link: https://cbi.iq/page/79. Repository name: Analysis of Financial Crowding Out in the Iraqi Economy for the Period 2004 – 2022– Appendix Dataset. https://doi.org/10.5281/zenodo.18404100 ^21^ This project contains the following extended data: Supplementary Appendix File (supporting tables and data used in the study) Data are available under the terms of the Creative Commons Attribution 4.0 licence (CC-BY 4.0).
