# Supplementary material for: Analysis of Financial Crowding Out in the Iraqi Economy for the Period 2004 – 2022
Source: F1000Res. 2026 Feb 10;15:223. [Version 1] doi: 10.12688/f1000research.173509.1 (PMC13150441; doi:10.12688/f1000research.173509.1)
Supplement: Peer review history

# Reviewer report: Reviewer response for version 1

**DOI:** 10.5256/f1000research.191333.r481239

**Published:** 2026-05-07

**Authors:** Maha Kalai

**Affiliations:** 1 University of Sfax, Sfax, Tunisia

**Academic Evaluation Report**

**Manuscript Title:** Analysis of Financial Crowding Out in the Iraqi Economy for the Period 2004–2022

**Authors:** Ahmed Jabbar & Basim Dikheel

**Journal:** F1000Research (Version 1; Peer Review Status: 1 Approved with Reservations)

**Report Prepared By:** Academic Reviewer

**Date:** May 4, 2026

**Executive Summary**

 This manuscript empirically investigates the financial crowding-out effect in Iraq by examining the dynamic relationship between government credit and private credit over the 2004–2022 period. Using time-series econometrics (ADF/PP unit root tests, Johansen-Juselius cointegration, VECM, Wald tests, and Granger causality), the authors find a statistically significant inverse relationship and unidirectional causality from government to private credit, consistent with monetary and Ricardian crowding-out theories. While the technical execution is competent and the dataset is publicly archived, the manuscript suffers from structural, narrative, and interpretive shortcomings that currently limit its scholarly and policy impact. A comprehensive revision is required before the article can be considered for full acceptance.

 The research article "Analysis of Financial Crowding Out in the Iraqi Economy for the Period 2004–2022" by Jabbar and Dikheel presents a methodologically sound econometric investigation into the inverse relationship between government and private credit in Iraq's rentier economy. The study demonstrates several notable strengths: the application of appropriate time-series techniques (ADF/PP unit root tests, Johansen-Juselius cointegration, VECM, and Wald causality tests) is correctly executed in EViews 12; the empirical findings robustly confirm a statistically significant short- and long-run inverse relationship consistent with monetary and Ricardian crowding-out theories; data transparency is commendable, with extended datasets archived on Zenodo under a CC-BY 4.0 license; and the graphical analysis effectively illustrates divergent credit trends, providing intuitive support for the quantitative results. Furthermore, the policy context, post-2004 fiscal expansion, chronic budget deficits, and oil-dependent revenues, is highly relevant to Iraq's macroeconomic challenges, lending practical urgency to the research question.

 While the technical execution is solid, several areas could be refined to enhance scholarly impact. The introduction could more sharply articulate the specific policy gap and research contribution relative to existing Iraqi crowding-out studies; the empirical model would be strengthened by a brief theoretical derivation linking the specification to established macroeconomic frameworks; and adding a concise discussion section to interpret key coefficients (e.g., the error-correction term's implied adjustment speed) in economic and institutional terms would deepen the analysis. Additionally, tightening the literature review to emphasize critical synthesis rather than descriptive summary, and deriving policy recommendations more explicitly from the empirical findings, would improve coherence and actionable relevance. These revisions are largely structural and interpretive rather than methodological, and do not undermine the validity of the core results.

**Decision: Minor Revision Recommended.**

 The manuscript's empirical foundation is robust, its topic is policy-relevant, and its technical execution meets standard econometric expectations. With targeted revisions to strengthen narrative framing, theoretical grounding, result interpretation, and policy linkage, without requiring additional data collection or re-estimation of models, the article will be well-positioned for indexing. It will make a valuable contribution to the literature on fiscal-monetary interactions in rentier economies.

Is the work clearly and accurately presented and does it cite the current literature?

Yes

If applicable, is the statistical analysis and its interpretation appropriate?

Yes

Are all the source data underlying the results available to ensure full reproducibility?

Yes

Is the study design appropriate and is the work technically sound?

Yes

Are the conclusions drawn adequately supported by the results?

Yes

Are sufficient details of methods and analysis provided to allow replication by others?

Partly

Reviewer Expertise:

Staistics and Economics

I confirm that I have read this submission and believe that I have an appropriate level of expertise to confirm that it is of an acceptable scientific standard.

# Reviewer report: Reviewer response for version 1

**DOI:** 10.5256/f1000research.191333.r480487

**Published:** 2026-05-07

**Authors:** Kamel Helali

**Affiliations:** 1 University of Sfax, Sfax, Tunisia

Report Review of F1000Research

**Title**: Analysis of Financial Crowding Out in the Iraqi Economy for the Period 2004 – 2022

**Authors:**

 - Ahmed Jabbar¹ (Financial and Banking Sciences, Al Iraqia University, Baghdad, Iraq)

 - Basim Dikheel² (Finance and Banking, University of Baghdad Al-Waziriya Campus, College of Administration and Economy, Baghdad, Iraq)

 Corresponding Author: Ahmed Jabbar (ahmed.al-khazraji@aliraqia.edu.iq)

**Journal**: F1000Research

**Manuscript Details:**

 - Dated: February 10, 2026

 - Version: 1

 - Article ID: 15:223

 - DOI: https://doi.org/10.12688/f1000research.173509.1

**Peer Review Status**: 1 approved with reservations (Reviewer: Malik Shahzad Shabbir, Ilma University, Karachi, Pakistan)

**Keywords**: Financial Crowding Out, Government Credit, Private Credit, Parkinson's Law, Financial Expulsion, Financial Attraction

 Study Period: 2004–2022 (monthly data)

 Data Source: Central Bank of Iraq (https://cbi.iq/page/79)

**Methodology:**

 - Descriptive, graphic, and statistical analysis using E-Views 12

 - Unit root tests: Augmented Dickey-Fuller (ADF) and Phillips-Perron (PP)

 - Cointegration analysis: Johansen-Juselius (1990) method

 - Vector Error Correction Model (VECM)

 - Wald test and Granger causality/block exogeneity tests

**Key Variables:**

 - Government Credit (GC)

 - Private Credit (CP)

**Main Findings:**

 1. Significant inverse relationship between government credit and private credit in both short and long terms

 2. Unidirectional causality from government credit to private credit

 3. Evidence supporting financial crowding out in the Iraqi economy

 4. Two-way cointegration relationship indicating long-term equilibrium dynamics

**Policy Recommendations:**

 - Transition from item-based to performance-oriented budgeting

 - Strengthen fiscal-monetary policy coordination

 - Enhance private sector participation through infrastructure development

 - Diversify revenue sources to reduce dependence on oil

**Data Availability:**

 - Underlying data: Central Bank of Iraq website

 - Extended data: Zenodo repository (https://doi.org/10.5281/zenodo.18404100)

**License**: Creative Commons Attribution 4.0 International (CC-BY 4.0)

 Competing Interests: None declared

 Funding: No grants involved

 This research article provides a timely and highly relevant examination of financial crowding out in the Iraqi economy over the critical post-2004 period. By focusing on the dynamic interplay between government and private credit, the authors successfully address a pressing macroeconomic challenge faced by rentier economies characterized by structural budget deficits, heavy reliance on oil revenues, and expansive public spending. The study’s clear articulation of the problem, grounded in Iraq's real-world economic vulnerabilities and political-economic transitions, establishes a strong foundation for its empirical investigation and underscores its significance for both academic discourse and economic policymaking in developing, resource-dependent nations.

 Methodologically, the paper demonstrates a rigorous and well-executed econometric framework. The authors appropriately employ a comprehensive suite of time-series techniques, including Augmented Dickey-Fuller and Phillips-Perron unit root tests, Johansen-Juselius cointegration analysis, and a Vector Error Correction Model (VECM) complemented by Wald and Granger causality tests. This methodological robustness ensures that the findings are statistically sound and resilient to common pitfalls associated with non-stationary macroeconomic data. Furthermore, the study's transparency is commendable; data are explicitly sourced from the Central Bank of Iraq, and extended datasets are made publicly available via a Zenodo repository, thereby enhancing reproducibility and scholarly credibility.

 The empirical results are presented with clarity and strongly support the study’s central hypothesis. The analysis convincingly identifies a significant inverse relationship and a unidirectional causal flow from government credit to private credit, effectively validating the presence of financial crowding out in Iraq. The authors adeptly contextualize these findings within established economic theories, thoughtfully contrasting Keynesian “crowding-in” perspectives with monetary and Ricardian explanations of private sector displacement. This theoretical anchoring, combined with precise statistical interpretation and logical economic intuition, adds substantial value to the literature on fiscal-monetary interactions and demonstrates a sophisticated understanding of macroeconomic dynamics in emerging markets.

 Beyond its empirical contributions, the article offers well-reasoned and actionable policy recommendations that directly stem from its findings. The call to transition from traditional item-based budgeting to performance-oriented frameworks, strengthen fiscal-monetary policy coordination, and actively foster private-sector participation through infrastructure development and revenue diversification provides a practical roadmap for Iraqi policymakers. Overall, this is a well-structured, methodologically sound, and policy-relevant study that meaningfully advances the understanding of financial crowding out. It serves as a valuable reference for researchers, economists, and decision-makers working on fiscal sustainability, debt management, and private-sector development in rentier and transitional economies.

**Peer Review Decision Recommendation**

**Recommendation: Accept with Minor Revisions**

**Overall Assessment**

 This manuscript presents a timely and empirically rigorous investigation of financial crowding out in Iraq's post-2004 economy. The study addresses a critical macroeconomic issue in rentier economies and employs appropriate econometric methodologies (ADF/PP unit root tests, Johansen-Juselius cointegration, VECM, Wald, and Granger causality tests) to examine the dynamic relationship between government and private credit. The findings are statistically sound, policy-relevant, and contribute meaningfully to the literature on fiscal-monetary interactions in developing, resource-dependent contexts.

**Major Strengths**

 1. Policy Relevance: The research tackles a pressing issue for Iraq and similar rentier economies, how public borrowing affects private sector access to finance, with clear implications for fiscal sustainability and private sector development.

 2. Methodological Rigor: The authors apply a comprehensive and appropriate time-series econometric framework, with transparent reporting of test statistics, critical values, and diagnostic checks.

 3. Data Transparency: Underlying data are sourced from the Central Bank of Iraq, and extended datasets are publicly archived via Zenodo, enhancing reproducibility and scholarly credibility.

 4. Theoretical Grounding: The paper effectively situates its empirical analysis within competing theoretical frameworks (Keynesian crowding-in vs. monetary/Ricardian crowding-out), demonstrating conceptual sophistication.

 5. Clear Policy Recommendations: Conclusions logically translate into actionable suggestions, including performance-based budgeting, fiscal-monetary coordination, and private sector infrastructure support.

**Suggestions for Minor Revision**

 1. Introduction Refinement: Strengthen the problem statement by explicitly articulating the policy gap this study fills and how its findings generalize (or do not) to other rentier economies.

 2. Literature Synthesis: Condense the theoretical review to highlight key debates and position the study's contribution more sharply against recent empirical work on crowding out in emerging markets.

 3. Economic Interpretation: Expand discussion of the VECM and causality results to clarify the economic intuition behind the estimated coefficients and their magnitude in practical terms.

 4. Limitations & Future Research: Add a brief subsection acknowledging data constraints (e.g., monthly aggregation, omitted variables) and suggesting avenues for extension (e.g., sectoral disaggregation, nonlinear thresholds).

 5. Title Precision: Consider refining the title to more precisely reflect the study's focus on financial (rather than investment) crowding out and its empirical scope.

**Final Recommendation**

 This is a well-executed, policy-relevant study that advances understanding of fiscal-financial dynamics in Iraq. With modest revisions to enhance contextual framing, theoretical positioning, and interpretive depth, the manuscript will be a valuable contribution to F1000Research and the broader literature on macroeconomic management in rentier states.

**Decision**: Accept with Minor Revisions

Is the work clearly and accurately presented and does it cite the current literature?

Yes

If applicable, is the statistical analysis and its interpretation appropriate?

Partly

Are all the source data underlying the results available to ensure full reproducibility?

Yes

Is the study design appropriate and is the work technically sound?

Yes

Are the conclusions drawn adequately supported by the results?

Yes

Are sufficient details of methods and analysis provided to allow replication by others?

Yes

Reviewer Expertise:

Econometrics and statistics

I confirm that I have read this submission and believe that I have an appropriate level of expertise to confirm that it is of an acceptable scientific standard.

# Reviewer report: Reviewer response for version 1

**DOI:** 10.5256/f1000research.191333.r468568

**Published:** 2026-04-08

**Authors:** Malik Shahzad Shabbir

**Affiliations:** 1 Ilma University, Karachi, Sindh, Pakistan

Dear Authors,

 You addressed the very interesting paper but still need more work to meet the journal requirement. I checked this title on Google Scholar and found a lot of articles publish in this domain. I am wondering from authors to see below published articles from well reputed journals and good scholars across the World. These studies are core area of your study. Will you justify how your study is different and important after reading these good studies, which published in high rank journals.

 Kindly see my comments below to address them to meet the indexing criteria of journal.

 Authors comments:

 1. Title needs to be revised as title and content of paper convey different meaning.

 2. The Introduction fails to motivate the study. In the present form, it resembles a mini-review of literature, rather than discussing any policy-level problem. Why this study is necessary? What policy level problem this study is addressing? How is the study expected to provide any solution to that problem? How does the choice of sample is complementing that problem? Are the results and policies generalizable? The introduction is silent in all these aspects. The mere choice of new variables, new methods, or choosing a new context is not considered as contribution of a study. 

 Refer to reference no. 1-3

 2. Literature review is extensively lengthy without any concrete conclusions. Try to find out the policy level problem persisting in the country from the third-party research reports, as academic literature will not be able to provide you with the specific policy issue. Kindly see below some of suggested studies.

 Refer to reference no. 4

 3. Materials and Methods

 How have the authors derived the empirical model? There should be a thorough theoretical underpinning behind the model. This section should be based on the logic of the authors, and citation/reference should appear here. This section will be followed by the empirical model.

 Authors have merely reported the results without discussing the economic intuitions behind the results. Are these results supporting or refuting the existing policies in the chosen context? Are the results directed towards any new policy initiatives?

 4. There is need to establish a discussion section and discussion of results should open up the threads of policy discussion, which is completely absent in this case. A mere comparison of the results doesn't ensure the novelty of the results unless they give out something new on the theory/policy front.

 Refer to reference no. 5-6

 5. The authors should summarize the results within a maximum of 3 sentences. Moreover, the policies should be comprehensive and practical in nature. The policies should be directly derived from the discussion of the results, and they should not go beyond the results. Moreover, theoretical implications and future research should be added in this section.

Is the work clearly and accurately presented and does it cite the current literature?

No

If applicable, is the statistical analysis and its interpretation appropriate?

No

Are all the source data underlying the results available to ensure full reproducibility?

No

Is the study design appropriate and is the work technically sound?

No

Are the conclusions drawn adequately supported by the results?

Partly

Are sufficient details of methods and analysis provided to allow replication by others?

Yes

Reviewer Expertise:

Green & Sustainable FinanceCorporate FinanceClimate ChangeGreen Energy

I confirm that I have read this submission and believe that I have an appropriate level of expertise to confirm that it is of an acceptable scientific standard, however I have significant reservations, as outlined above.

## References

[1] : Exploring tail reliance and interconnectedness: GCC stock markets, sustainable investments, and renewable energy cryptos through Q-VAR analysis. *Natural Resource Modeling, 38, e12411* .2025;

[2] : Nexus among health expenditures, ICTs and sustainable economic growth: Insights from South Asian Nationals health and economic issues. *The European Journal of Health Economics* .2025;10.1007/s10198-025-01799-y40658344

[3] : Dynamic prospective life cycle assessment of transition paths for the Austrian steel industry. *Energy, Sustainability and Society* .2026;

[4] : Assessing the impact of economic volatility on renewable energy tokens, GCC stock indices, and green assets. *Environment, Development and Sustainability* .2025; 10.1007/s10668-025-06325-x 10.1007/s10668-025-06325-x

[5] : Does natural resources matter? Nexus among renewable energy policies, technological innovation, environmental protection, and economic growth. *Energy Strategy Reviews* .2024;51: 10.1016/j.esr.2023.101272 10.1016/j.esr.2023.101272

[6] : Examining the Impact of Energy Policies on CO2 Emissions with Information and Communication Technologies and Renewable Energy. *Studies in Nonlinear Dynamics & Econometrics* .2024;28(3) : 10.1515/snde-2022-0065 545-552 10.1515/snde-2022-0065
